# Hashimoto’s Encephalopathy in a Critically Ill Patient With Polysubstance Use: A Diagnostic Challenge

**DOI:** 10.1016/j.aed.2025.07.005

**Published:** 2025-07-16

**Authors:** Shifa Akhter, Mario Gonzalez, Raghav Gupta, Jose I. Gonzalez

**Affiliations:** 1 Department of Internal Medicine, North Alabama Medical Center, Florence, Alabama; 2 Department of Internal Medicine, North Delhi Municipal Corporation Medical College, Malka Ganj, Delhi, India; 3 Department of Internal Medicine, Universidad Autonoma de Centro America, Puntarenas Province, Paso Canoas, Costa Rica

**Keywords:** Hashimoto’s encephalopathy, autoimmune encephalopathy, steroid-responsive encephalopathy, antithyroid antibodies, toxic-metabolic encephalopathy

## Abstract

**Background/Objective:**

Hashimoto’s encephalopathy (HE) is a rare autoimmune condition associated with high titers of antithyroid antibodies and a variable neuropsychiatric presentation. Diagnosing HE is often challenging, particularly in critically ill patients with confounding etiologies such as polysubstance use.

**Presentation:**

A case of a 57-year-old male is presented with a history of hypertension and chronic tobacco use that was found unresponsive at home. Emergency Medical Services found him obtunded, hypoxic, and hypertensive; he was intubated and admitted to the intensive care unit. Laboratory evaluation revealed rhabdomyolysis, acute kidney injury, hyperkalemia, and a positive urine drug screen for amphetamines, opioids, benzodiazepines, and marijuana. Despite supportive management, his encephalopathy persisted. Extensive workup, including autoimmune and infectious panels, revealed elevated antithyroid peroxidase and antithyroglobulin antibodies, supporting the diagnosis of HE. HIV testing and cluster of differentiation 4 counts were negative, ruling out AIDS-related encephalopathy and progressive multifocal leukoencephalopathy. High-dose corticosteroids were initiated, resulting in significant neurologic improvement. He was later transitioned to oral hydrocortisone and levothyroxine.

**Conclusion:**

This case underscores the importance of pursuing early autoimmune testing in cases of encephalopathy with unclear etiology. HE remains a diagnosis of exclusion, a consideration that should only arise after comprehensive evaluation and treatment of potential metabolic, toxic, structural, and infectious causes have failed to yield improvement. In this case, timely recognition allowed for an appropriate corticosteroid trial and subsequent clinical response, reaffirming the critical need to include HE in the differential when other common causes have been ruled out.

**Highlights**

• Hashimoto’s encephalopathy (HE) should be considered in cases of unexplained encephalopathy when all reversible causes have been treated or ruled out
• Thyroid function may be normal in HE; antibody testing is essential to confirm the diagnosis. Steroid responsiveness is a key diagnostic and therapeutic feature of HE
• Toxicologic, metabolic, and autoimmune causes may coexist and complicate the clinical picture
• A multidisciplinary and evolving diagnostic approach is critical for critically ill patients with prolonged altered mental status

**Clinical Relevance**

This case underscores the necessity of considering autoimmune encephalopathy in critically ill patients with prolonged altered mental status. Early recognition of Hashimoto’s encephalopathy and prompt corticosteroid therapy can result in significant neurologic recovery, even when initial presentations are complicated by polysubstance use and metabolic disturbances.

## Introduction

Hashimoto's encephalopathy (HE), also known as steroid-responsive encephalopathy associated with autoimmune thyroiditis, is a rare and potentially reversible autoimmune condition characterized by a wide spectrum of neuropsychiatric manifestations, including seizures, confusion, altered mental status, and coma. Despite increasing recognition, its pathogenesis remains incompletely understood.^1^ Elevated antithyroid antibodies, particularly (antithyroid peroxidase) anti-TPO and antithyroglobulin antibodies, are commonly observed in HE patients; however, their pathogenic role is debated. Recent literature suggests that these antibodies may serve more as markers of systemic autoimmunity rather than direct mediators of neurologic injury, given their prevalence in the general population (10% to 30%) and inconsistent correlation with disease severity.^2^

Diagnosing HE is challenging due to its heterogeneous clinical presentation and the absence of specific diagnostic markers. The condition often mimics other encephalopathies, such as toxic-metabolic, infectious, or other autoimmune encephalitides, necessitating a diagnosis of exclusion. Furthermore, the variability in neuroimaging and electroencephalogram findings adds to the diagnostic complexity. A high index of clinical suspicion and a thorough diagnostic workup are essential for the accurate identification of HE.^2^

This report discusses a critically ill patient initially presumed to have methamphetamine-induced encephalopathy, but further evaluation led to the diagnosis of HE. This case underscores the importance of considering HE in the differential diagnosis of unexplained encephalopathy, particularly when standard evaluations fail to yield a definitive cause.

## Report

A case is presented of a 57-year-old Caucasian male with a history of untreated hypertension, cigarette smoking, and polysubstance abuse, including intravenous (IV) drug use. According to the patient’s partner, he had reportedly been experiencing gastrointestinal symptoms, including nausea, vomiting, and diarrhea, for the past 2 days, along with an associated headache and photophobia. He had no reported history of fever, chills, or focal neurological deficits. His last known well time was the night prior to his presentation in the Emergency Department (ED). However, in the morning, the patient did not wake up at his usual time and was found unresponsive with blood around his nose. Emergency Medical Services were called, and according to their assessment, the patient was minimally responsive to painful stimulus with an oxygen saturation of 57%, heart rate in the 150s, and a blood pressure of 165/100 mmHg. There was no evidence of bowel or bladder incontinence. Naloxone was administered without clinical response, and he was transported to the ED.

In the ED, the patient was unresponsive with a Glasgow Coma Scale (GCS) score of 6 (E1, V1, M4), blood pressure of 200/120 mmHg, and heart rate of 128 bpm. He was intubated for airway protection and admitted to the intensive care unit. Laboratory findings were notable for leukocytosis, severe rhabdomyolysis (creatine kinase >15 000 U/L), acute kidney injury (AKI) (creatinine 4.0 mg/dL), hyperkalemia (potassium 6.4 mmol/L), and metabolic acidosis (arterial pH 7.15). Urine toxicology was positive for amphetamines, opioids, fentanyl, benzodiazepines, and marijuana. Computed tomography (CT) and CT angiography of the head and neck showed no acute abnormalities. Electroencephalogram revealed diffuse encephalopathic changes without seizure activity. Chest imaging demonstrated right upper lobe nodules.

He was managed for suspected toxic-metabolic encephalopathy secondary to polysubstance use and hypertensive emergency. Treatment included endotracheal intubation, aggressive IV hydration, IV labetalol for blood pressure control, empiric antibiotics (vancomycin and piperacillin-tazobactam), and hyperkalemia management with IV calcium gluconate, insulin, and dextrose.

After 24 h, while still sedated, the patient showed intermittent arousability but did not follow commands (GCS 9: E3, V1, M5). At 48 h, he completed spontaneous awakening and breathing trials, with an improvement in GCS to 13–14 (E4, V4, M5), and was subsequently extubated. Postextubation, while he continued to demonstrate clinical improvement, he remained lethargic, disoriented, and was unable to follow commands during the motor exam consistently.

Around day 5, a Neurology consultation prompted further evaluation, including a magnetic resonance imaging scan of the brain, a lumbar puncture, thyroid function tests, autoimmune markers, and infectious disease screening. Magnetic resonance imaging demonstrated bilateral white matter hyperintensities and basal ganglia involvement, raising concern for HIV-related immunodeficiency and progressive multifocal leukoencephalopathy (PML) in the context of IV drug use. However, HIV testing and cluster of differentiation 4 counts were normal, effectively ruling out PML. Cerebrospinal fluid analysis showed mildly elevated protein; cerebrospinal fluid infectious and autoimmune panels were negative.

Thyroid studies revealed elevated TPO antibodies (319 IU/mL; normal <35), elevated thyroglobulin antibodies (9.4 IU/mL), and thyroid-stimulating hormone of 7.9 μIU/mL, raising suspicion for HE. The patient was started on IV methylprednisolone 500 mg daily for possible steroid-responsive encephalopathy. Over the next 48 h, he demonstrated marked improvement in alertness, orientation, and ability to follow simple motor commands. He was transitioned to oral hydrocortisone and levothyroxine.

Creatine kinase levels normalized, and renal function gradually recovered; he was then transferred to the medical floor for ongoing c are. The patient briefly underwent physical therapy, which was later discontinued as he declined to participate. The patient also declined to be discharged to a rehabilitation center. He was eventually discharged with outpatient endocrinology follow-up. A timeline outlining the patient's neurological progression and key clinical interventions is summarized in Table.

**Table:** Timeline of Neurological Status and Key Interventions During Hospitalization

Day	Key events
1	- GCS 6–7 (E1, V1, M4) - Intubated for airway protection due to altered mental status - Managed for toxic-metabolic encephalopathy secondary to polysubstance use and hypertensive emergency
3	- GCS improved to 13 (E4, V4, M5) - Neurological exam: Confused, drowsy, able to follow simple commands - Successfully extubated
5	- GCS stable at 13 (E4, V4, M5) - Neurological exam: Persistently lethargic, with slow speech and minimal interaction, inconsistently followed commands - Ordered workup including brain MRI, lumbar puncture, thyroid studies, autoimmune and infectious disease panels
6	- GCS stable at 13 (E4, V4, M5) - Neurological status unchanged - With infectious, metabolic, and structural etiologies excluded, empiric high-dose intravenous corticosteroids were initiated based on elevated TSH and TPO antibodies
8	- GCS improved to 15 (E4, V5, M6) - Neurological exam: Alert, oriented to person, place, and time; followed motor commands appropriately - Transferred from ICU to medical ward - Initiated ambulation up to 4-5 feet with moderate assistance in physical therapy
10	- GCS stable at 15 (E4, V5, M6) - Neurological status stable - Transitioned to oral corticosteroids and levothyroxine - The patient declined physical therapy. - Discharged

Abbreviation: GCS =Glasgow Coma Scale; ICU = Intensive Care Unit; MRI = magnetic resonance imaging; TPO = thyroid peroxidase; TSH = thyroid-stimulating hormone.

## Discussion

This case highlights the diagnostic challenges associated with autoimmune encephalopathies, particularly in patients presenting with complex and confounding clinical pictures. HE remains a diagnosis of exclusion, typically supported by the presence of antithyroid antibodies and an improved clinical response to corticosteroid therapy. Although anti-TPO antibody levels in reported cases of HE are often markedly elevated—frequently in the high hundreds or thousands—even moderately increased levels, such as the 319 IU/mL observed in this patient, may be clinically significant when aligned with supportive clinical features and after alternative etiologies have been ruled out.^3^ The patient’s rapid neurological improvement following the initiation of corticosteroids further reinforced the presumptive diagnosis of HE.^4^

In this patient, the initial working diagnosis was methamphetamine-induced seizure or toxic encephalopathy. The presence of rhabdomyolysis, AKI, hyperkalemia, and hypertensive emergency was consistent with stimulant toxicity. However, the persistence of encephalopathy despite the correction of metabolic disturbances and the absence of acute findings on neuroimaging prompted further diagnostic evaluation. A broad differential diagnosis was considered and systematically investigated.

• Substance-induced toxic encephalopathy and seizure: Strongly suspected, given the positive urine toxicology screen and clinical presentation. The patient was managed supportively, including use of the Clinical Institute Withdrawal Assessment for Alcohol protocol.
• Alcohol or benzodiazepine withdrawal: Empiric treatment was initiated with thiamine, folate, and benzodiazepines due to the potential for withdrawal syndromes contributing to altered mental status.
• Sepsis-associated encephalopathy: Considered based on leukocytosis, elevated procalcitonin, and gastrointestinal symptoms. However, blood and urine cultures remained negative, and antibiotics were subsequently discontinued.
• Metabolic encephalopathy: Metabolic derangements, including acidosis, hyperkalemia, and AKI, were believed to contribute to altered mental status but did not fully account for the persistent encephalopathy following correction.
• Hepatic encephalopathy: Mild transaminitis was noted, but the absence of hyperammonemia and other clinical signs of liver failure made this diagnosis unlikely.
• Central nervous system infection: Magnetic resonance imaging findings and the patient's IV drug abuse history prompted evaluation for infectious causes, including HIV-associated opportunistic infections such as PML. HIV and cluster of differentiation 4 testing were negative. Lumbar puncture revealed mildly elevated protein levels and herpes simplex virus polymerase chain reaction was negative.
• Autoimmune encephalitis (nonthyroid): Paraneoplastic and autoimmune encephalitis panels were negative, with the exception of elevated antithyroid antibodies.
• Vascular etiologies: Stroke, intracranial hemorrhage, and posterior reversible encephalopathy syndrome were ruled out with non-contrast CT and computed tomography of the head and neck.

Ultimately, the presence of elevated TPO and thyroglobulin antibodies, in conjunction with a marked clinical response to corticosteroid therapy, supported the diagnosis of HE. It is possible that the patient’s seizures and rhabdomyolysis were precipitated or exacerbated by the underlying autoimmune process. Early recognition of HE is critical, as timely initiation of corticosteroids can significantly impact the clinical trajectory and improve outcomes.^5^ The coexistence of polysubstance use further complicated the clinical picture. Fentanyl and benzodiazepine positivity likely contributed to central nervous system depression, necessitating mechanical ventilation.

This case underscores the importance of including autoimmune encephalopathies, such as HE, in the differential diagnosis of patients with persistent altered mental status despite correction of metabolic, toxic, infectious, and structural abnormalities. HE remains a diagnosis of exclusion and should only be considered after thorough evaluation has ruled out more common etiologies. Prompt autoimmune screening and empiric corticosteroid therapy may be lifesaving and are associated with significantly improved patient outcomes.^6^

Beyond diagnostic considerations, recent literature advocates for the combined use of the Coma Recovery Scale–Revised and the GCS to more accurately evaluate consciousness levels in patients with disorders of consciousness.^7^ The Coma Recovery Scale–Revised offers enhanced sensitivity for detecting subtle neurologic changes, facilitating reliable monitoring and supporting the initiation of individualized rehabilitation plans. Early rehabilitation, particularly in patients with autoimmune encephalopathies requiring intensive care, should not be overlooked. As demonstrated in Intensive Care Unit populations with disorders of consciousness, early mobilization plays a critical role in preventing disuse atrophy, joint contractures, and secondary complications while also promoting neuroplasticity and cognitive recovery. The structured framework described by Magro et al,^7^ comprising early assessment, goal setting, individualized intervention, and continuous monitoring, can be adapted to support functional recovery and minimize long-term disability in patients with HE. Incorporating physical and occupational therapy during the subacute phase, even when patients remain partially obtunded, aligns with this proactive rehabilitation model and may contribute to improved clinical outcomes.

## Patient Consent

Informed consent was not required as all patient data were fully anonymized and no identifiable information is included in this case report.

## Disclosure

The authors have no conflicts of interest to disclose.
